# Supplementary material for: Management of the Adverse Effects of Immune Checkpoint Inhibitors
Source: Vaccines (Basel). 2020 Oct 1;8(4):575. doi: 10.3390/vaccines8040575 (PMC7711557; doi:10.3390/vaccines8040575)
Supplement: Supplementary file 1 [file vaccines-08-00575-s001.pdf]

Table S1- Frequency of adverse effects of immune checkpoint inhibitors (ICPI).

ICPI (Trade name)	Affected organ or system of the human body	Immune-related adverse effects						
		Adverse reactions observed	(Frequency)					
			Very common	Common	Uncommon	Rare	Very rare	Unknown
**Ipilimumab[a] (Yervoy) [1]**	**Infections and infestations**	Sepsis, septic shock, urinary tract infection, respiratory tract infection			X			
	**Neoplasms benign, malignant and unspecified (including cysts and polyps)**	Tumour pain		X				
		Paraneoplastic syndrome			X			
	**Blood and lymphatic system disorders**	Anaemia, lymphopenia		X				
		Haemolytic anaemia, thrombocytopenia, eosinophilia, neutropenia			X			
		Histiocytosis haematophagic						X
	**Immune system disorders**	Hypersensitivity			X			
		Anaphylactic reaction					X	
	**Endocrine disorders**	Hypopituitarism (including hypophysitis), hypothyroidism		X				
		Adrenal insufficiency, secondary adrenocortical insufficiency, hyperthyroidism, hypogonadism			X			
		Autoimmune thyroiditis, thyroiditis				X		
	**Metabolism and nutrition disorders**	Decreased appetite	X					
		Dehydration, hypokalemia		X				

		Hyponatremia, alkalosis, hypophosphatemia, tumour lysis syndrome, hypocalcaemia				X		
	**Psychiatric disorders**	Confusional state		X				
		Mental status changes, depression, decreased libido			X			
	**Nervous system disorders**	Peripheral sensory neuropathy, dizziness, headache, lethargy		X				
		Guillain-Barré syndrome, meningitis (aseptic), autoimmune central neuropathy (encephalitis), syncope, cranial neuropathy, brain oedema, peripheral neuropathy, ataxia, tremor, myoclonus, dysarthria			X			
		Myasthenia gravis				X		
	**Eye disorders**	Blurred vision, eye pain		X				
		Uveitis, vitreous haemorrhage, iritis, eye oedema, blepharitis, reduced visual acuity, foreign body sensation in eyes, conjunctivitis			X			
		Vogt-Koyanagi-Harada syndrome, serous retinal detachment				X		
	**Cardiac disorders**	Arrhythmia, atrial fibrillation			X			
	**Vascular disorders**	Hypotension, flushing, hot flush		X				
		Vasculitis, angiopathy, peripheral ischaemia, orthostatic hypotension			X			
		Temporal arteritis				X		
	**Respiratory, thoracic and mediastinal disorders**	Dyspnea, cough		X				
		Respiratory failure, acute respiratory distress syndrome, lung infiltration, pulmonary oedema, pneumonitis, allergic rhinitis			X			
	**Gastrointestinal disorders**	Diarrhoea, vomiting, nausea	X					

		Gastrointestinal haemorrhage, colitis, constipation, gastroesophageal reflux disease, abdominal pain, mucosal inflammation		X				
		Gastrointestinal perforation, large intestine perforation, intestinal perforation, peritonitis, gastroenteritis, diverticulitis, pancreatitis, enterocolitis, gastric ulcer, large intestinal ulcer, stomatitis, oesophagitis, ileus			X			
		Proctitis				X		
	**Hepatobiliary disorders**	Abnormal hepatic function		X				
		Hepatic failure, hepatitis, hepatomegaly, jaundice			X			
	**Skin and subcutaneous tissue disorders**	Rash, pruritus	X					
		Dermatitis, erythema, vitiligo, urticaria, eczema, alopecia, night sweats, dry skin		X				
		Toxic epidermal necrolysis, leukocytoclastic vasculitis, skin exfoliation, hair colour changes			X			
		Erythema multiforme, psoriasis, drug reaction with eosinophilia and systemic symptoms (DRESS)				X		
		Pemphigoid						X
	**Musculoskeletal and connective tissue disorders**	Arthralgia, myalgia, musculoskeletal pain, muscle spasms		X				
		Polymyalgia rheumatica, myositis, arthritis, muscular weakness			X			
		Polymyositis				X		
	**Renal and urinary disorders**	Renal failure, glomerulonephritis, autoimmune nephritis, renal tubular acidosis, haematuria			X			

		Proteinuria				X		
	**Reproductive system and breast disorders**	Amenorrhea			X			
	**General disorders and administration site conditions**	Fatigue, injection site reaction, pyrexia	X					
		Chills, asthenia, oedema, pain, influenza-like illness		X				
		Multi-organ failure, systemic inflammatory response syndrome, infusion related reaction			X			
**Pembrolizumab [b] (Keytruda) [2]**	**Infections and infestations**	Pneumonia		X				
	**Blood and lymphatic system disorders**	Anaemia	X					
		Thrombocytopaenia, lymphopaenia		X				
		Neutropaenia, leukopaenia, eosinophilia			X			
		Immune thrombocytopenic purpura, haemolytic anaemia, pure red cell aplasia, haemophagocytic lymphohistiocytosis				X		
	**Immune system disorders**	Infusion-related reaction		X				
		Sarcoidosis			X			
		Solid organ transplant rejection						X
	**Endocrine disorders**	Hypothyroidism	X					
		Hyperthyroidism		X				
		Adrenal insufficiency, hypophysitis, thyroiditis			X			
	**Metabolism and nutrition disorders**	Decreased appetite	X					
		Hyponatraemia, hypokalaemia, hypocalcaemia		X				
		Type 1 diabetes mellitus			X			
	**Psychiatric disorders**	Insomnia		X				

	**Nervous system disorders**	Headache	X					
		Dizziness, neuropathy peripheral, lethargy, dysgeusia		X				
		Epilepsy			X			
		Encephalitis, Guillain-Barré syndrome, myelitis, myasthenic syndrome, meningitis (aseptic)				X		
	**Eye disorders**	Dry eye		X				
		Uveitis			X			
		Vogt-Koyanagi-Harada syndrome				X		
	**Cardiac disorders**	Cardiac arrhythmia (including atrial fibrillation)		X				
		Pericardial effusion, pericarditis			X			
		Myocarditis				X		
	**Vascular disorders**	Hypertension		X				
	**Respiratory, thoracic and mediastinal disorders**	Dyspnoea, cough	X					
		Pneumonitis		X				
	**Gastrointestinal disorders**	Diarrhoea, abdominal pain, nausea, vomiting, constipation	X					
		Colitis, dry mouth		X				
		Pancreatitis, gastrointestinal ulceration			X			
		Small intestinal perforation				X		
	**Hepatobiliary disorders**	Hepatobiliary disorders			X			
	**Skin and subcutaneous tissue disorders**	Rash, pruritus	X					
		Severe skin reactions, erythema, dry skin, vitiligo, eczema, alopecia, dermatitis acneiform		X				
		Lichenoid keratosis, psoriasis, dermatitis, papule, hair colour changes			X			

		Toxic epidermal necrolysis, Stevens-Johnson syndrome, erythema nodosum				X		
	**Musculoskeletal and connective tissue disorders**	Musculoskeletal pain, arthralgia	X					
		Pain in extremity, myositis, arthritis		X				
		Tenosynovitis			X			
	**Renal and urinary disorders**	Nephritis, acute kidney injury		X				
		Nephritis			X			
	**General disorders and administration site conditions**	Fatigue, asthenia, oedema, pyrexia	X					
		Influenza-like illness, chills		X				
**Nivolumab** [b] **(Opdivo)** [3]	**Infections and infestations**	Upper respiratory tract infection		X				
		Pneumonia, bronchitis			X			
		Aseptic meningitis						X
	**Neoplasms benign, malignant and unspecified (including cysts and polyps)**	Histiocytic necrotising lymphadenitis (Kikuchi lymphadenitis)				X		
	**Blood and lymphatic system disorders**	Neutropaenia	X					
		Eosinophilia			X			
		Haemophagocytic lymphohistiocytosis						X
	**Immune system disorders**	Infusion related reaction, hypersensitivity		X				
		Anaphylactic reaction			X			
		Solid organ transplant rejection, sarcoidosis						X

	**Endocrine disorders**	Hypothyroidism, hyperthyroidism		X				
		Adrenal insufficiency, hypopituitarism, hypophysitis, thyroiditis, diabetes mellitus			X			
		Diabetic ketoacidosis				X		
		Hypoparathyroidism						X
	**Metabolism and nutrition disorders**	Decreased appetite		X				
		Dehydration, metabolic acidosis			X			
		Tumour lysis syndrome						X
	**Nervous system disorders**	Peripheral neuropathy, headache, dizziness		X				
		Polyneuropathy, autoimmune neuropathy (including facial and abducens nerve paresis)			X			
		Guillain-Barré syndrome, demyelination, myasthenic syndrome, encephalitis				X		
	**Eye disorders**	Uveitis, blurred vision, dry eye			X			
		Vogt-Koyanagi-Harada syndrome						X
	**Cardiac disorders**	Tachycardia, pericardial disorders			X			
		Arrhythmia (including ventricular arrhythmia), atrial fibrillation, myocarditis				X		
	**Vascular disorders**	Hypertension		X				
		Vasculitis			X			
	**Respiratory, thoracic and mediastinal disorders**	Pneumonitis, dyspnoea, cough		X				
		Pleural effusion			X			
		Lung infiltration				X		
	**Gastrointestinal disorders**	Diarrhoea, nausea	X					

		Colitis, stomatitis, vomiting, abdominal pain, constipation, dry mouth		X				
		Pancreatitis, gastritis			X			
		Duodenal ulcer				X		
	**Hepatobiliary disorders**	Hepatitis			X			
		Cholestasis				X		
	**Skin and subcutaneous tissue disorders**	Rash, pruritus	X					
		Vitiligo, dry skin, erythema, alopecia		X				
		Erythema multiforme, psoriasis, rosacea, urticaria			X			
		Toxic epidermal necrolysis, Stevens-Johnson syndrome				X		
	**Musculoskeletal and connective tissue disorders**	Musculoskeletal pain, arthralgia		X				
		Polymyalgia rheumatica, arthritis			X			
		Sjogren's syndrome, myopathy, myositis (including polymyositis), rhabdomyolysis				X		
	**Renal and urinary disorders**	Tubulointerstitial nephritis, renal failure (including acute kidney injury)			X			
	**General disorders and administration site conditions**	Fatigue	X					
		Pyrexia, oedema (including peripheral oedema)		X				
		Pain, chest pain			X			
**Atezolizumab (Tecentriq) [4]**	**Infections and infestations**	Urinary tract infection	X					
	**Blood and lymphatic system disorders**	Thrombocytopenia		X				
	**Immune system disorders**	Infusion-related reaction		X				

	**Endocrine disorders**	Hypothyroidism		X				
		Hyperthyroidism, diabetes mellitus, adrenal insufficiency			X			
		Hypophysitis				X		
	**Metabolism and nutrition disorders**	Decreased appetite	X					
		Hypokalaemia, hyponatraemia, hyperglycemia		X				
	**Nervous system disorders**	Guillain-Barré syndrome, meningoencephalitis			X			
		Myasthenic syndrome				X		
	**Eye disorders**	Uveitis				X		
	**Vascular disorders**	Hypotension		X				
	**Cardiac disorders**	Myocarditis				X		
	**Respiratory, thoracic and mediastinal disorders**	Dyspnoea, cough	X					
		Pneumonitis, hypoxia, nasal congestion, nasopharyngitis		X				
	**Gastrointestinal disorders**	Diarrhoea, vomiting, nausea	X					
		Abdominal pain, colitis, dysphagia, oropharyngeal pain		X				
		Pancreatitis			X			
	**Hepatobiliary disorders**	Aspartate aminotransferase increased or alanine aminotransferase increased, hepatitis		X				
	**Skin and subcutaneous tissue disorders**	Rash, pruritus	X					
		Psoriasis			X			
	**Musculoskeletal and connective tissue disorders**	Arthralgia, back pain, musculoskeletal pain	X					
		Myositis			X			
	**Renal and urinary disorders**	Nephritis				X		
		Pyrexia, fatigue, asthenia	X					

	**General disorders and administration site conditions**	Influenza like illness, chills		X				
**Avelumab** [b, c] **(Bavencio) [5]**	**Blood and lymphatic system disorders**	Anaemia	X					
		Lymphopenia		X				
		Thrombocytopenia, eosinophilia			X			
	**Immune system disorders**	Drug hypersensitivity, hypersensitivity anaphylactic reaction, Type I hypersensitivity			X			
	**Endocrine disorders**	Hypothyroidism		X				
		Adrenal insufficiency, hyperthyroidism, thyroiditis, autoimmune thyroiditis, adrenocortical insufficiency acute, autoimmune hypothyroidism, hypopituitarism			X			
	**Metabolism and nutrition disorders**	Decreased appetite	X					
		Type 1 diabetes mellitus			X			
	**Nervous system disorders**	Headache, dizziness, neuropathy peripheral		X				
		Guillain-Barré Syndrome			X			
	**Eye disorders**	Uveitis			X			
	**Cardiac disorders**	Myocarditis				X		
	**Vascular disorders**	Hypertension, hypotension		X				
		Flushing			X			
	**Respiratory, thoracic and mediastinal disorders**	Cough, dyspnoea	X					
		Pneumonitis		X				
	**Gastrointestinal disorders**	Nausea, diarrhoea, constipation, vomiting, abdominal pain	X					

		Dry mouth		X				
		Colitis, autoimmune colitis, enterocolitis, ileus			X			
		Pancreatitis				X		
	**Hepatobiliary disorders**	Autoimmune hepatitis, acute hepatic failure, hepatic failure, hepatitis			X			
	**Skin and subcutaneous tissue disorders**	Rash, pruritus, rash maculo-papular, dry skin		X				
		Rash pruritic, erythema, rash generalized, psoriasis, rash erythematous, rash macular, rash papular, dermatitis exfoliative, erythema multiforme, pemphigoid, pruritus generalized, eczema, dermatitis			X			
	**Musculoskeletal and connective tissue disorders**	Back pain, arthralgia	X					
		Myalgia		X				
		Myositis			X			
	**Renal and urinary disorders**	Tubulo-interstitial nephritis			X			
	**General disorders and administrative site conditions**	Fatigue, pyrexia, oedema peripheral	X					
		Asthenia, chills, influenza like illness		X				
		Systemic inflammatory response syndrome			X			
**Durvalumab[d] (Imfinzi) [6]**	**Infections and infestations**	Upper respiratory tract infections, pneumonia	X					
		Dental and oral soft tissue infections, oral candidiasis, influenza		X				
	**Endocrine disorders**	Hypothyroidism	X					
		Hyperthyroidism		X				
		Adrenal insufficiency, type 1 diabetes mellitus			X			
		Hypophysitis/hypopituitarism, diabetes insipidus				X		

	**Nervous System Disorders**	Myasthenia gravis, meningitis				X		
		Noninfective encephalitis, Guillain-Barre syndrome						X
	**Cardiac disorders**	Myocarditis				X		
	**Respiratory, thoracic and mediastinal disorders**	Cough/productive cough, pneumonitis	X					
		Dysphonia		X				
		Interstitial lung disease			X			
	**Gastrointestinal disorders**	Diarrhoea, abdominal pain	X					
		Colitis		X				
	**Hepatobiliary disorders**	Aspartate aminotransferase increased or alanine aminotransferase increased		X				
		Hepatitis			X			
	**Skin and subcutaneous tissue disorders**	Rash, pruritus	X					
		Dermatitis, night sweats		X				
		Pemphigoid			X			
	**Musculoskeletal and connective tissue disorders**	Myalgia		X				
		Myositis			X			
		Polymyositis				X		
	**Renal and urinary disorders**	Blood creatinine increased, dysuria		X				
		Nephritis			X			
	**General disorders and administration site conditions**	Pyrexia	X					
		Peripheral oedema		X				
	**Immune system disorders**	Infusion-related reaction		X				

**Cemiplimab (Libtayo) [7]**		Sjogren's syndrome, immune thrombocytopenic purpura, vasculitis			X			
		Solid organ transplant rejection						X
	**Endocrine disorders**	Hypothyroidism, hyperthyroidism		X				
		Type 1 diabetes mellitus, adrenal insufficiency, hypophysitis, thyroiditis			X			
	**Nervous system disorders**	Paraneoplastic encephalomyelitis, chronic inflammatory demyelinating polyradiculoneuropathy, encephalitis, meningitis, Guillain-Barre syndrome, central nervous system inflammation, neuropathy peripheral, myasthenia gravis			X			
	**Eye disorders**	Keratitis			X			
	**Cardiac disorders**	Myocarditis, pericarditis			X			
	**Respiratory, thoracic and mediastinal disorders**	Pneumonitis, dyspnoea,		X				
	**Gastrointestinal disorders**	Diarrhoea,	X					
		Stomatitis		X				
	**Hepatobiliary disorders**	Hepatitis		X				
	**Skin and subcutaneous skin disorders**	Rash, pruritus	X					
	**Musculoskeletal and connective tissue disorders**	Arthralgia, musculoskeletal pain, arthritis		X				
		Muscular weakness, polymyalgia rheumatica			X			
		Myositis				X		
	**Renal and urinary disorders**	Nephritis			X			

	**General disorders and administration site conditions**	Fatigue	X					

a-Patients with advanced melanoma, b-Monotherapy, c-Results from a phase I study in solid tumors, d-Patients with locally advanced unresectable non-small cell lung cancer.

[1] Summary of product characteristics Yervoy. Available online: https://www.ema.europa.eu/en/documents/product-information/yervoy-epar-product-information_en.pdf (accessed on 20 September 2020)

[2]Summary of product characteristics Keytruda. Available online: https://www.ema.europa.eu/en/documents/product-information/keytruda-epar-product_information_en.pdf (accessed on 20 September 2020)

[3] Summary of product characteristics Opdivo. Available online: https://www.ema.europa.eu/en/documents/product-information/opdivo-epar-product-information_en.pdf (accessed on 20 September 2020)

[4] Summary of product characteristics Tecentriq. Available online: https://www.ema.europa.eu/en/documents/product-information/tecentriq-epar-product-information_en.pdf (accessed on 20 September 2020)

[5] Summary of product characteristics Bavencio. Available online: https://www.ema.europa.eu/en/documents/product-information/bavencio-epar-product-information_en.pdf (accessed on 20 September 2020)

[6] Summary of product characteristics Imfinzi. Available online: https://www.ema.europa.eu/en/documents/product-information/imfinzi-epar-product-information_en.pdf (accessed on 20 September 2020)

[7] Summary of product characteristics Libtayo. Available online: https://www.ema.europa.eu/en/documents/product-information/libtayo-epar-product-information_en.pdf (accessed on 20 September 2020)
